# DNA-PKcs inhibitors sensitize neuroendocrine tumor cells to peptide receptor radionuclide therapy *in vitro* and *in vivo*

**DOI:** 10.7150/thno.82963

**Published:** 2023-05-21

**Authors:** Thom G.A. Reuvers, Nicole S. Verkaik, Debra Stuurman, Corrina de Ridder, Marian C. Clahsen-van Groningen, Erik de Blois, Julie Nonnekens

**Affiliations:** 1 Department of Molecular Genetics, Erasmus MC Cancer Institute, Erasmus University Medical Center Rotterdam, The Netherlands.; 2 Department of Radiology and Nuclear Medicine, Erasmus MC Cancer Institute, Erasmus University Medical Center Rotterdam, The Netherlands.; 3 Department of Urology, Erasmus University Medical Center Rotterdam, The Netherlands.; 4 Department of Pathology, Erasmus University Medical Center Rotterdam, The Netherlands.; 5 Institute of Experimental Medicine and Systems Biology, RWTH Aachen University, Aachen, Germany.

**Keywords:** Neuroendocrine tumors, peptide receptor radionuclide therapy, DDR inhibitors, DNA-PKcs, ionizing radiation

## Abstract

**Background:** Peptide receptor radionuclide therapy (PRRT) increases progression-free survival and quality of life of neuroendocrine tumor (NET) patients, however complete cures are rare and dose-limiting toxicity has been reported. PRRT induces DNA damage of which DNA double strand breaks (DSBs) are the most cytotoxic. DNA-dependent protein kinase catalytic subunit (DNA-PKcs) is a key player in DSB repair and its inhibition therefore is a potential way to enhance PRRT efficacy without increasing the dosage.

**Methods:** We analyzed effects of combining PRRT and DNA-PKcs inhibitor AZD7648 on viability, cell death and clonogenic survival on SSTR2-expressing cell lines BON1-SSTR2, GOT1 and NCI-H69. Therapy-induced DNA damage response was assessed by analyzing DSB foci levels and cell cycle distributions. *In vivo* efficacy was investigated in BON1-SSTR2 and NCI-H69 xenografted mice and hematologic and renal toxicity were monitored by blood counts, creatinine levels and analyzing renal morphology.

**Results:** Combining PRRT and AZD7648 significantly decreased viability of BON1-SSTR2, GOT1 and NCI-H69 cells and induced cell death in GOT1 and BON1-SSTR2 cells. A strong effect of AZD7648 on PRRT-induced DSB repair was found. In GOT1 cells, this was accompanied by induction of cell cycle blocks. However, BON1-SSTR2 cells were unable to fully arrest their cell cycle and polyploid cells with high DNA damage levels were detected. *In vivo*, AZD7648 significantly sensitized BON1-SSTR2 and NCI-H69 xenograft models to PRRT. In addition, combination therapy did not induce significant changes in body weight, blood composition, plasma creatinine levels and renal morphology, indicating the absence of severe acute hematologic and renal toxicity.

**Conclusion:** These results highlight that the potentiation of the therapeutic effect of PRRT by DNA-PKcs inhibition is a highly effective and well-tolerated therapeutic strategy. Based on our findings, we recommend initiation of phase I/II studies in patients to find a safe and effective combination regimen.

## Introduction

NETs are a relatively rare form of cancer originating from the diffuse neuroendocrine system 1. PRRT using [^177^Lu]Lu-[DOTA-Tyr^3^]octreotate (^177^Lu-DOTA-TATE), was approved as a treatment for somatostatin receptor subtype 2 (SSTR2)-positive advanced gastroenteropancreatic NETs (GEP-NETs). Unfortunately, complete cure with the current regimen of PRRT is rare and dose-limiting kidney and bone marrow toxicity has been reported 2, 3. These challenges have sparked research interests into PRRT radiobiology to better understand cellular effects and eventually improve therapy 4. After systemic administration, ^177^Lu-DOTA-TATE binds to SSTR2 on NET cells, after which radioactive decay of lutetium-177 induces DNA damage leading to cancer cell death 5, 6. However, cells can repair this damage by employing a tightly orchestrated network of proteins called the DNA damage response (DDR), which counteracts the anti-cancer effects of PRRT and consequently can lead to cancer cell survival 7. An attractive avenue of improvement is therefore to combine PRRT with targeted inhibitors of DDR proteins, as has been demonstrated using inhibitors of poly (ADP-ribose) polymerase 1/2 (PARP1/2), a protein central in the repair of DNA single-strand breaks 8, 9. The strategy of combining radiation with other DDR inhibitors has been used extensively for external beam radiation therapy (EBRT), but due to the inherent differences between the physical aspects of EBRT and PRRT (e.g. timing, dose rate, radiation source), these methods cannot readily be transferred to PRRT 10.

One of the most lethal DNA lesions induced by radiation therapies are double-strand breaks (DSBs). Therefore, inhibition of the DSB repair machinery has been evaluated in a vast panel of cancer types as radiosensitization strategy. The major DSB repair pathway is non-homologous end-joining (NHEJ) and a central protein in this pathway is DNA-PKcs 11. Cells derived from DNA-PKcs-deficient mice display severe radiosensitivity, highlighting the importance of this protein in genomic stability and DNA repair 12-14. Moreover, upregulation of DNA-PKcs has been shown to correlate with radioresistance in cancer cells 15-17. Interestingly, functions other than in DSB repair have been found for DNA-PKcs, including transcription regulation, telomere maintenance and cell cycle progression, and those may play additional roles in the response to radiation therapies 18. For these reasons, different small molecule inhibitors of DNA-PKcs have been developed the past years, with varying selectivity and potency 19. These have been shown to significantly sensitize different tumor types to EBRT 20-22. However, data on the effect of these inhibitors combined with PRRT is still lacking.

In this paper we investigated PRRT radiosensitization of NET in *in vitro* and *in vivo* models by DNA-PKcs inhibition and showed that selective DNA-PKcs inhibition is a feasible strategy to potentiate the cancer-killing effect of PRRT.

## Materials & Methods

### Cell culture and reagents

BON1-SSTR2 cells were generated by transfecting wildtype BON1 cells with the pcDNA3.1-SSTR2 vector as described previously 23, using Lipofectamine Transfection Reagent (ThermoFisher Scientific). Selection was done for 1 week using 2 mg/mL G418 (Invivogen), after which cells were cultured in presence of 500 μg/mL G418 every other passage. BON1-SSTR2 cells were cultured in DMEM/F12 medium (Gibco), supplemented with 10% fetal calf serum (FCS; Biowest) and 1% penicillin-streptomycin (PS; Sigma-Aldrich). GOT1 cells were maintained in RPMI 1640 (Gibco), supplemented with 10% FCS, 1% PS, 5 mM L-glutamine (Stemcell Technologies), 5 µg/mL insulin (Sigma) and 5 µg/mL human holo-transferrin (Sigma). NCI-H69 cells (ATCC) were cultured in RPMI 1640, supplemented with 10% FCS and 1% PS. All cells were cultured at 37 °C and 5% CO_2_.

### Radiolabeling

Lu-Mark was purchased from IDB Holland and ^177^Lu-DOTA-TATE for *in vitro* studies was synthesized in-house according to a standard labeling procedure as used for patient treatment (molar activity 53 MBq/nmol, radiochemical yield > 98% and radiochemical purity > 95%). For *in vivo* studies, ^177^Lu-DOTA-TATE was synthesized in-house at a molar activity of 86 MBq/nmol, radiochemical yield of > 95% and radiochemical purity of > 90%.

### Antibodies

For immunofluorescent (IF) and immunohistochemical (IHC) stainings, primary antibodies were used for SSTR2 (Abcam, ab134152, 1:250) and 53BP1 (Novus Biologicals, NB100-904, 1:1000). The used secondary antibodies were goat-anti-rabbit IgG Alexa Fluor 488 (Thermo Fisher, A-11006, 1:1000) for IF and peroxidase-conjugated AffiniPure donkey-anti-rabbit IgG (Jackson ImmunoResearch, 705-035-147, 1:100) for IHC.

For western blotting, primary antibodies were used for DNA-PKcs (homemade 24, 1:1000), and phospho-DNA-PKcs (Ser2056) (CST, E9J4G, 1:1000). The used secondary antibody was peroxidase-conjugated AffiniPure donkey-anti-rabbit IgG (Jackson ImmunoResearch, 705-035-147, 1:1000).

### Viability assay

Cells were treated with ^177^Lu-DOTA-TATE or vehicle in suspension (1 MBq/mL (1.9x10^-8^ M) in culture medium, 2x10^5^ cells/mL) and plated in triplicate in white-walled, round, flat bottom polystyrene 96-well plates (Corning) in the presence of a concentration range of AZD7648 in 200 µL culture medium per well (BON1-SSTR2: 500 cells/well; GOT1: 3x10^4^ cells/well; NCI-H69: 1.5x10^4^ cells/well). Samples were incubated for 7 days before readout. At the time of readout, 100 µL of CellTiterGlo^®^ 2.0 assay reagent (Promega) was added to each well, after which plates were incubated for 10 min at RT. Luminescence was recorded using a Spectramax iD3x microplate reader (Molecular Devices) without wavelength selection. All raw luminescence values were normalized to the values of cells untreated with AZD7648 to calculate cell viability relative to vehicle- or PRRT-treated controls. Experiments were performed as 3 independent replicates.

### Colony survival assay

Following ^177^Lu-DOTA-TATE (1 MBq/mL (1.9x10^-8^ M) in culture medium, 6x10^5^ cells in 60mm-dish) or vehicle treatment for 2 h, BON1-SSTR2 cells were seeded in 6-well plates at a density of 300 cells/well in triplicate in the continuous presence of an AZD7648 concentration range. Plates were incubated for 14 days to allow colony formation. After incubation, the medium was removed, wells were washed in phosphate-buffered saline (PBS; Lonza), and colonies were fixed and stained in 50% (v/v) methanol (Sigma), 7% (v/v) acetic acid (Sigma) and 0.1% (m/v) Brilliant Blue R (Sigma) in dH_2_O. Colonies were quantified manually. Experiments were performed as 3 independent replicates.

### Cell death detection

GOT1 and BON1-SSTR2 cells were treated with ^177^Lu-DOTA-TATE or vehicle in suspension (1 MBq/mL (1.9x10^-8^ M) in culture medium, 2x10^5^ cells/mL) and seeded with and without 1 μM AZD7648 in 6-well plates. After 5 days, cell death detection was done using the CellEvent™ Caspase-3/7 Green Flow Cytometry Assay Kit (Invitrogen), following the manufacturer's instructions. In short, cells were incubated with CellEvent Caspase3/7 Green Detection Reagent (500 nM) in culture medium for 55 min at RT in the dark, after which SYTOX™ AADvanced™ Dead Cell Stain (1 μM) was added for an additional 5 min. Fluorescent signal of both dyes was detected by flow cytometry on a LSRFortessa flow cytometer (BD Biosciences). Gating and data analysis were performed using FlowJo™ software (BD Biosciences). Experiments were performed as 3 independent replicates.

### Cell cycle distributions

GOT1 and BON1-SSTR2 cells were treated with ^177^Lu-DOTA-TATE or vehicle in suspension (1 MBq/mL (1.9x10^-8^ M) in culture medium, 2x10^5^ cells/mL) and incubated with and without 1 μM AZD7648 for 3 days, after which 5-ethynyl-2'-deoxyuridine (EdU; ThermoFisher Scientific) was added for 3 h at 30 μM until fixation in ice-cold ethanol. Cells were permeabilized in PBS + 0.1% Triton X-100 (Merck) on ice. For EdU detection, samples were incubated with a reaction mix consisting of 40 mM Tris buffer, 4 mM CuSO_4_ (Sigma), 30 μM Atto488 azide (ATTO-TEC) and 4 mM ascorbic acid (Sigma) for 30 min at RT in the dark. Samples were washed in PBS + 1% bovine serum albumin (BSA; Sigma) and afterwards resuspended in 1 µg/mL DAPI (Thermo Fisher Scientific) in PBS until analysis. EdU- and DAPI-intensities were recorded on a LSRFortessa flow cytometer. Data analysis was done using FlowJo™ software. Experiments were performed as 3 independent replicates.

### DNA damage analysis and nuclear area quantification

After ^177^Lu-DOTA-TATE or vehicle treatment in suspension (1 MBq/mL (1.9x10^-8^ M) in culture medium, 2x10^5^ cells/mL), GOT1 and BON1-SSTR2 cells were incubated with and without 1 μM AZD7648 for 24, 72 and 120 h. For GOT1, cells were plated in 24-well plates, trypsinized and cytospun on glass coverslips using a Rotofix 32A centrifuge (Hettich). For BON1-SSTR2, after PRRT cells were plated on glass coverslips. Cells were fixed in 2% paraformaldehyde (PFA) for 15 min at RT at the indicated timepoints.

To stain cells for 53BP1, samples were permeabilized in PBS + 0.1% Triton X-100. Cells were washed in blocking buffer (PBS + 0.5% BSA) and incubated with primary antibody (diluted in blocking buffer) for 1 h at RT in the dark. Subsequently, samples were incubated with secondary antibody (diluted in blocking buffer) for 1 h at RT in the dark. Coverslips were mounted on microscope slides using Vectashield containing DAPI (Vector Laboratories).

Imaging of 53BP1 foci was done using 405 nm (DAPI) and 488 nm (53BP1) lasers on a TCS SP5 confocal microscope (Leica) with an oil immersion 40x objective. At least four fields of view were captured and 50 cells were analyzed per condition. Images were acquired as Z-stacks. Creation of maximum Z-projections of each image, nuclei segmentation and analysis of the number of 53BP1 foci per nucleus was done in FIJI using a homemade macro. In short, nuclei were segmented in the maximum Z-projections based on thresholding the DAPI channel with a defined minimum and maximum nucleus size. Clusters of cells that could not be segmented correctly were removed from the analysis. Additionally, nuclear size was determined based on this segmentation method. 53BP1 foci were segmented per nucleus by thresholding the Alexa Fluor 488-channel, again with a defined minimum and maximum focus size. Experiments were performed as 3 independent replicates.

### *In vivo* experiments

Animal experiments were approved by the Animal Welfare Committee of the Erasmus Medical Center Rotterdam and were conducted in compliance with European guidelines. 6-week-old Rj:NMRI-*Foxn1^nu/nu^* mice (Janvier Labs) were subcutaneously injected in the right flank with 5x10^6^ BON1-SSTR2 cells in Matrigel (33% v/v; Corning) in Hank's Balanced Salt Solution (HBSS; Gibco) or 5x10^6^ NCI-H69 cells in HBSS. Tumor size was determined by caliper measurements. When tumors reached an average size of 200-400 mm^3^, mice were randomly distributed in treatment groups and intravenously injected in the tail vein with 30 MBq (0.35 nmol) ^177^Lu-DOTA-TATE or with vehicle (PBS + 0.1% BSA) (N=9 per group). Starting 2 h before ^177^Lu-DOTA-TATE treatment, mice received AZD7648 (50 mg/kg or 100 mg/kg) or vehicle (0.5% hydroxypropyl methylcellulose (HPMC; Colorcon)/ 0.1% Tween-80 (Sigma-Aldrich) by oral gavage daily for 7 consecutive days. Tumor volume and body weight were determined twice per week. When the tumor volume reached 2000 mm^3^, blood was collected by orbital puncture and mice were euthanized by cervical dislocation.

At the timepoint of euthanization, whole blood samples were collected in EDTA tubes, after which white and red blood cell and platelet counts were performed using an abc Plus counter (scil Vet). Blood plasma was isolated in lithium-heparin tubes and plasma creatinine levels were determined using a Cobas 8000 analyzer (Roche). Tumor and kidneys were collected immediately after euthanization and processed as described below.

### Tumor and kidney SSTR2- and histology assessment

Tumor and kidneys were collected and fixed in 10% formalin (J.T.Baker) for 24 h at RT and embedded in paraffin. Subsequently, 4 μm sections were generated. For the tumors, hematoxylin & eosin (H&E) staining was performed using standard procedures 6. For IHC analysis of SSTR2, sections were deparaffinized and rehydrated and antigen retrieval was performed in Tris-EDTA buffer (pH 9.0). Slides were incubated with 3% H_2_O_2_ in methanol and subsequently blocked in 5% protifar (Nutricia) in washing buffer (PBS + 0.1% Triton X-100). Sections were incubated with primary antibody in blocking buffer at 4 °C O/N, washed and subsequently incubated with secondary antibody in blocking buffer for 30 min. Antibodies were detected using the DAB+ Substrate Chromogen Kit (DAKO). Samples were mounted using Pertex Mounting Medium.

For the kidneys, periodic acid-schiff (PAS) staining was carried out on a Ventana BenchMark Special Stains machine (Roche) as previously described 25. Assessment of kidney damage was performed in a blinded manner by scoring acute tubular necrosis (ATN) on 10x magnification. Every section was scored for tubular dilatation, cast deposition, brush border loss and necrosis. Scoring was done by a 5-point scale with a score of 0 reflecting virtually no damage, and 5 reflecting severe damage.

Visualization of all stainings described in this section (H&E, IHC, PAS) was done by a NanoZoomer slice scanner (Hamamatsu Photonics).

### Statistical analyses

Statistical analyses for all experiments were performed in Graphpad Prism (version 8). Used statistical tests include one-way ANOVA with Tukey's test for multiple comparisons. All statistical analyses yielding a p-value < 0.05 were considered significant.

## Results

### SSTR2-overexpression in BON1 cells increases ^177^Lu-DOTA-TATE binding

As availability of human cell lines of neuroendocrine origin with sufficiently high SSTR2-expression is limited 26, we generated a SSTR2-overexpressing clone of the neuroendocrine cell line BON1. Compared to the relatively low SSTR2-expression of wildtype BON1 cells, a sharp increase in SSTR2-levels was confirmed by microscopy and flow cytometry in BON1-SSTR2 cells (Figure S1A-B). Functional analysis was done by uptake studies, which confirmed an approximately 100-fold increase in binding of ^177^Lu-DOTA-TATE to BON1-SSTR2 cells compared to wildtype (Figure S1C). The majority of the radionuclide, 84%, was internalized after a 2 h-incubation period, as expected for an agonist.

### Inhibition of DNA-PKcs by AZD7648 sensitizes SSTR2-expressing NET *in vitro* models to PRRT

For our studies on DNA-PKcs inhibition, we used AZD7648, a recently developed potent and selective inhibitor of DNA-PKcs catalytic activity 22. Inhibition of DNA-PKcs autophosphorylation at Ser2056 (p-DNA-PKcs), which is induced by ionizing radiation and required for DSB repair 27, was confirmed after AZD7648 treatment in BON1-SSTR2 cells (Figure S2A). Compared to PRRT monotherapy, AZD7648 treatment did not decrease p-DNA-PKcs levels after combination therapy. An explanation for this is that a high amount of DNA-PKcs is activated upon increased levels of DNA damage during combination therapy, and that this activation is only partially inhibited at 1 μM AZD7648. To further confirm the effects of AZD7648 on DNA-PKcs activity, we measured the balance between classical non-homologous end joining (C-NHEJ) and microhomology-mediated end-joining (MMEJ) in BON1-SSTR2 cells using an end-joining assay (Figure S2B). Inhibition of C-NHEJ is expected to lead to an increase of MMEJ as one of the compensatory repair mechanisms and indeed, we found that 1 μM of AZD7648 led to a severe shift to MMEJ (91%) in BON1-SSTR2, compared to 24% of relative MMEJ activity in untreated cells, confirming the inhibitory effects of AZD7648 on C-NHEJ repair.

**Figure 1:**
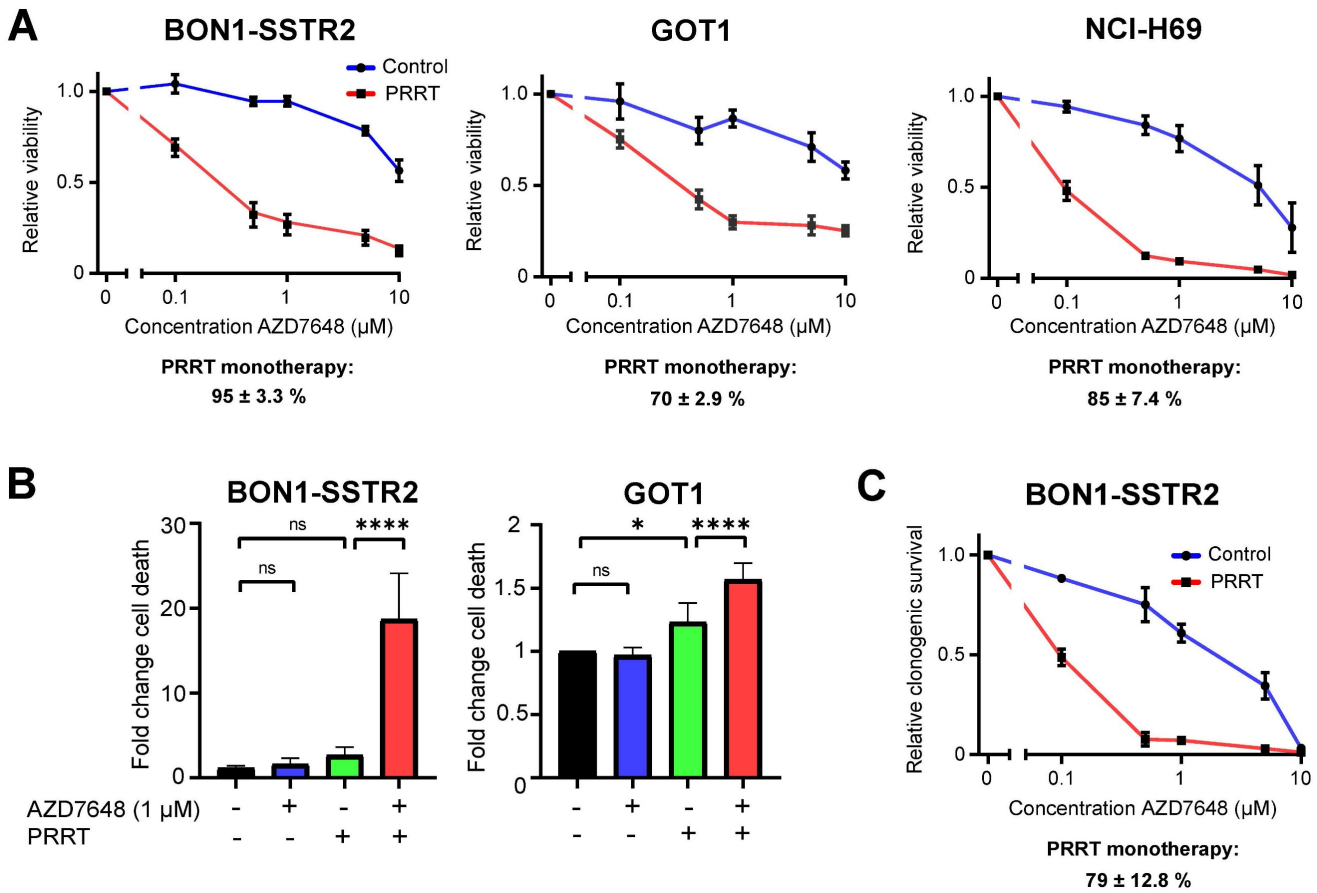
***In vitro* effects of PRRT + AZD7648 on SSTR2-positive cell lines. A,** Effect of AZD7648 on cell viability of BON1-SSTR2, GOT1 and NCI-H69 cells administered as monotherapy (blue line) or combined with 1 MBq/mL PRRT for 4 h (red line). Both curves are normalized to their respective viability without AZD7648**.** Relative viability (mean ± SD) after PRRT monotherapy, as compared to vehicle, is indicated below the corresponding graph for each cell line. **B,** Cell death (apoptosis/necrosis) induction at 5 days post-PRRT for BON1-SSTR2 and GOT1 cells, expressed as fold change from untreated control. Cells were treated with 1 μM AZD7648. Ns = not significant; *p > 0.05; ****p > 0.0001. **C,** Effect of AZD7648 on clonogenic survival of BON1-SSTR2 cells administered as monotherapy (blue line) or combined with 1 MBq/mL PRRT (red line). Both curves are normalized to their respective survival without AZD7648**.** Relative clonogenic survival (mean ± SD) after PRRT monotherapy, as compared to vehicle, is indicated below the corresponding graph. All data points represent the mean of 3 independent replicates. All error bars represent SD.

To determine the effect of combining PRRT and AZD7648 on cell viability, we treated SSTR2-positive cell lines BON1-SSTR2, GOT1 and NCI-H69 with PRRT or vehicle and a concentration range of AZD7648. For all three cell lines, AZD7648 induced a notable concentration-dependent decrease in cell viability in PRRT-treated cells compared to vehicle-treated cells, indicating that DNA-PKcs inhibition significantly sensitizes these cells to PRRT (Figure 1A). To validate if the enhanced effect of combination therapy on cell viability was due to cell death, apoptosis and necrosis induction was measured at 5 days post-PRRT in GOT1 and BON1-SSTR2 cells, two cell lines from our panel that are derived from neuroendocrine tumors (Figure 1B; Figure S3). PRRT + AZD7648 significantly increased cell death levels compared to control in GOT1 cells (1.6-fold) compared to PRRT alone (1.2-fold). In BON1-SSTR2 cells, a massive induction of cell death was detected after combination therapy (15-fold increase compared to control) as compared to PRRT monotherapy (2.2-fold). No induction of cell death was seen in the AZD7648 monotherapy-treated cells. Finally, to further confirm the observed therapy effects on cell viability, we assessed clonogenic survival after therapy in BON1-SSTR2 cells (Figure 1C). Due to its low proliferation rate, this assay was not feasible for GOT1 cells. Again, AZD7648 dose-dependently decreased the number of BON1-SSTR2 colonies after PRRT versus vehicle-treated cells.

### Combination of PRRT and AZD7648 results in high levels of DNA damage and induces cell type-dependent effects on cell cycle

**Figure 2:**
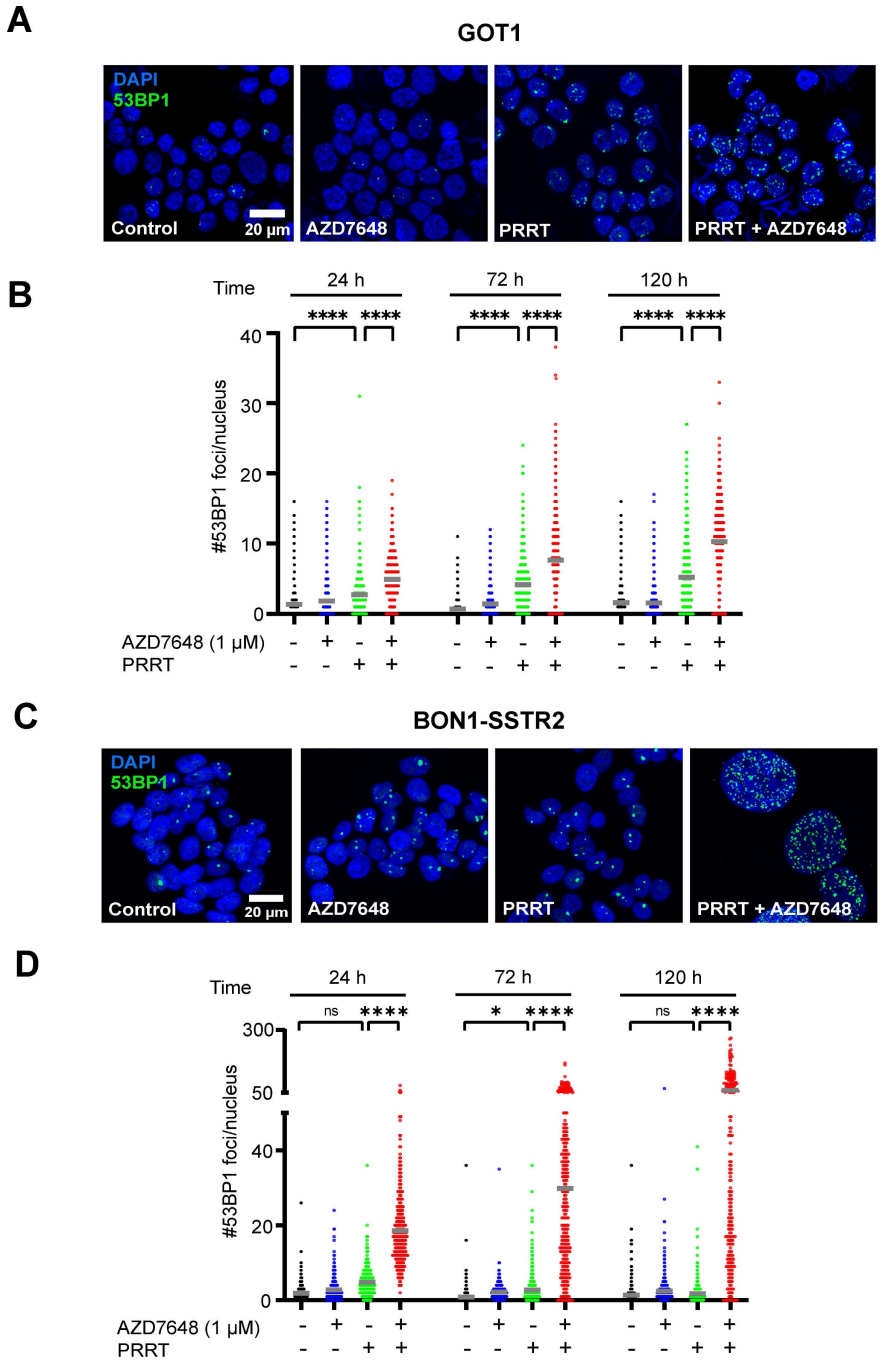
** Effect of PRRT + AZD7648 on DSB repair foci.** Cells were treated with 1 MBq/mL PRRT or vehicle and subsequently with 1 μM AZD7648 or vehicle. **A+C,** Representative images of IF staining for 53BP1 (green) as a marker for DSBs in GOT1 cells (A) and BON1-SSTR2 cells (C) after vehicle treatment, AZD7648 or PRRT monotherapy and PRRT + AZD7648. DAPI was used as a nuclear counterstain. Images are shown for 120 h post-PRRT. **B+D,** Quantification of the number of 53BP1 foci per nucleus for GOT1 (B) and BON1-SSTR2 (D) cells at 24, 72 and 120 h post-PRRT treatment. At least 50 nuclei in 3 independent experiments were analyzed per condition. Graphs show all data points from these 3 experiments merged. Grey horizontal bars represent the mean. Ns = not significant; *p > 0.05; ****p > 0.0001.

As DNA-PKcs plays a central role in DSB repair, we measured the number of 53BP1 foci (a marker for DSBs 28) per nucleus in GOT1 and BON1-SSTR2 cells after treatment. Quantification of the number of foci in GOT1 showed a small but significant increase for PRRT alone, as compared to control (Figure 2A-B), while only at 72 h post-PRRT a significant increase in number of foci could be detected in BON1-SSTR2 (Figure 2C-D). However, when PRRT was combined with AZD7648, a significant increase in foci number was seen for both cell lines at 24, 72 and 120 h after PRRT. For BON1-SSTR2, this was accompanied by the presence of large nuclei containing a massive number of foci **(**Figure 2C-D). No increase in number of 53BP1 foci was seen for AZD7648 monotherapy.

**Figure 3:**
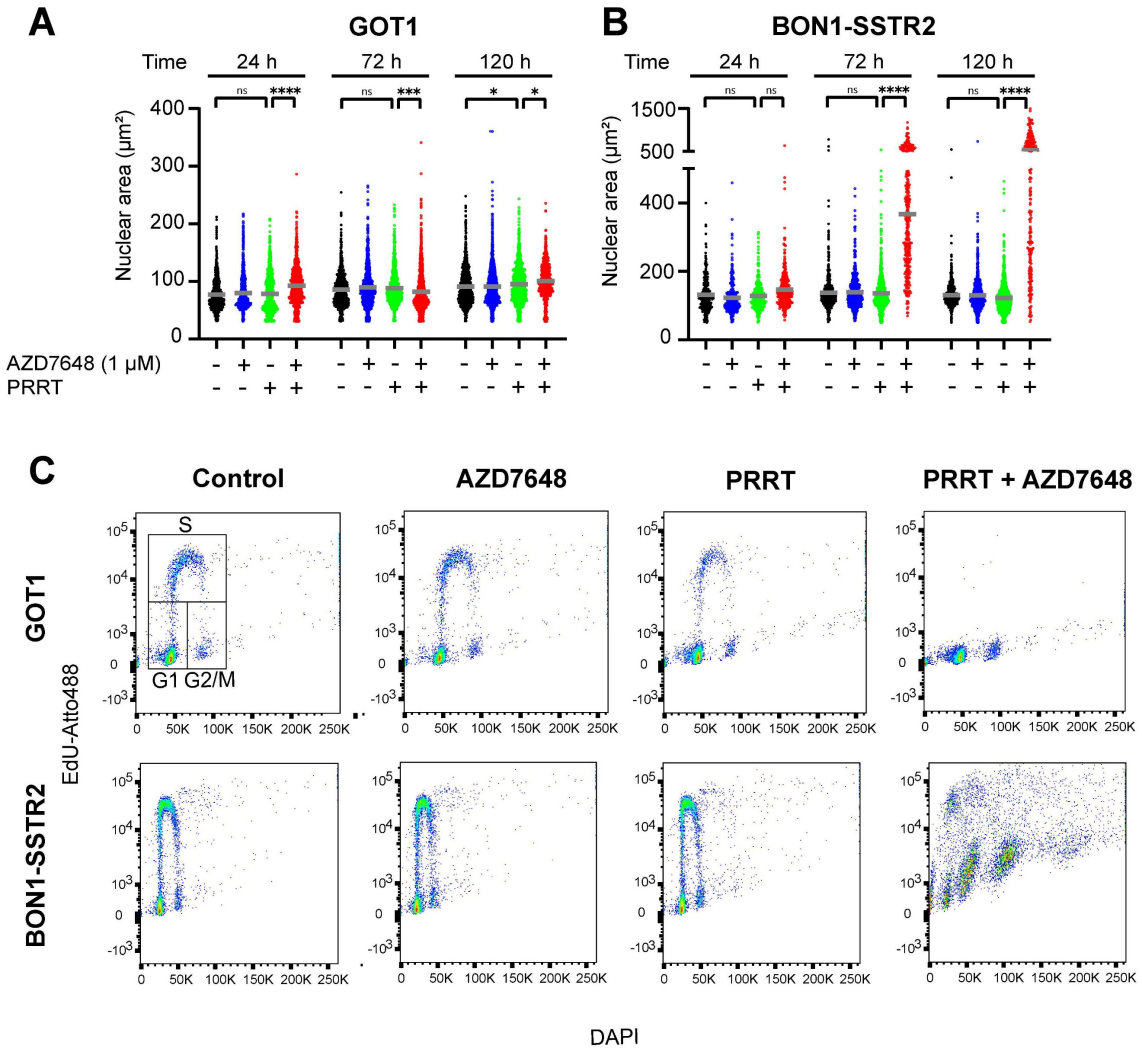
** Cell type-dependent effects of PRRT + AZD7648 on cell cycle.** Cells were treated with 1 MBq/mL PRRT or vehicle and subsequently with 1 μM AZD7648 or vehicle.** A+B,** Quantification of nuclear area for BON1-SSTR2 (A) or GOT1 (B) at 24, 72 and 120 h after post-PRRT. At least 50 nuclei in 3 independent experiments were analyzed per condition. Graphs show all data points from these 3 experiments merged. Grey horizontal bars represent the mean. Ns = not significant; *p>0.05; ***p>0.001; ****p>0.0001. **C+D,** Cell cycle distribution analysis for GOT1 (upper panel) and BON1-SSTR2 (lower panel). Cells were analyzed for DNA content (DAPI, x-axis) and EdU incorporation (EdU-Atto488, y-axis). An example of the gating strategy for G1-, S- and G2/M-phases of the cell cycle is indicated in the GOT1 control sample plot. Scatter plot colors indicate the density of events. Experiment was performed as 3 independent replicates and results from 1 representative experiment are shown.

To investigate the observed effects on the nucleus in more detail, the nuclear area was quantified for both cell lines. For GOT1, no trend in differences in nuclear size could be detected after both monotherapies or combination therapy over time post-PRRT (Figure 3A), while for BON1-SSTR2, it was confirmed that PRRT + AZD7648 induced a sharp and significant increase in nuclear size (Figure 3B), which further increased over time. Subsequently, we assessed therapy-induced cell cycle distribution by DNA content and EdU-incorporation analysis in both cell lines (Figure 3C; Figure S4). In GOT1 cells, PRRT alone slightly decreased the percentage of cells with EdU-incorporation, indicating a decrease of S-phase cells and induction of cell cycle blocks. PRRT + AZD7648 further abolished proliferating cells in S-phase compared to PRRT alone. In contrast, neither AZD7648 nor PRRT monotherapy significantly changed the cell cycle distribution of BON1-SSTR2 cells. However, PRRT + AZD7648 induced cells with large DNA content, while a relatively high number of S-phase cells could still be detected. This indicated that BON1-SSTR2 cells could only partially induce cell cycle blocks and that polyploidy was induced after combination therapy, confirming a difference in cellular response compared to GOT1.

### AZD7648 potentiates PRRT anti-tumor effects in SSTR2-expressing xenograft models

**Figure 4:**
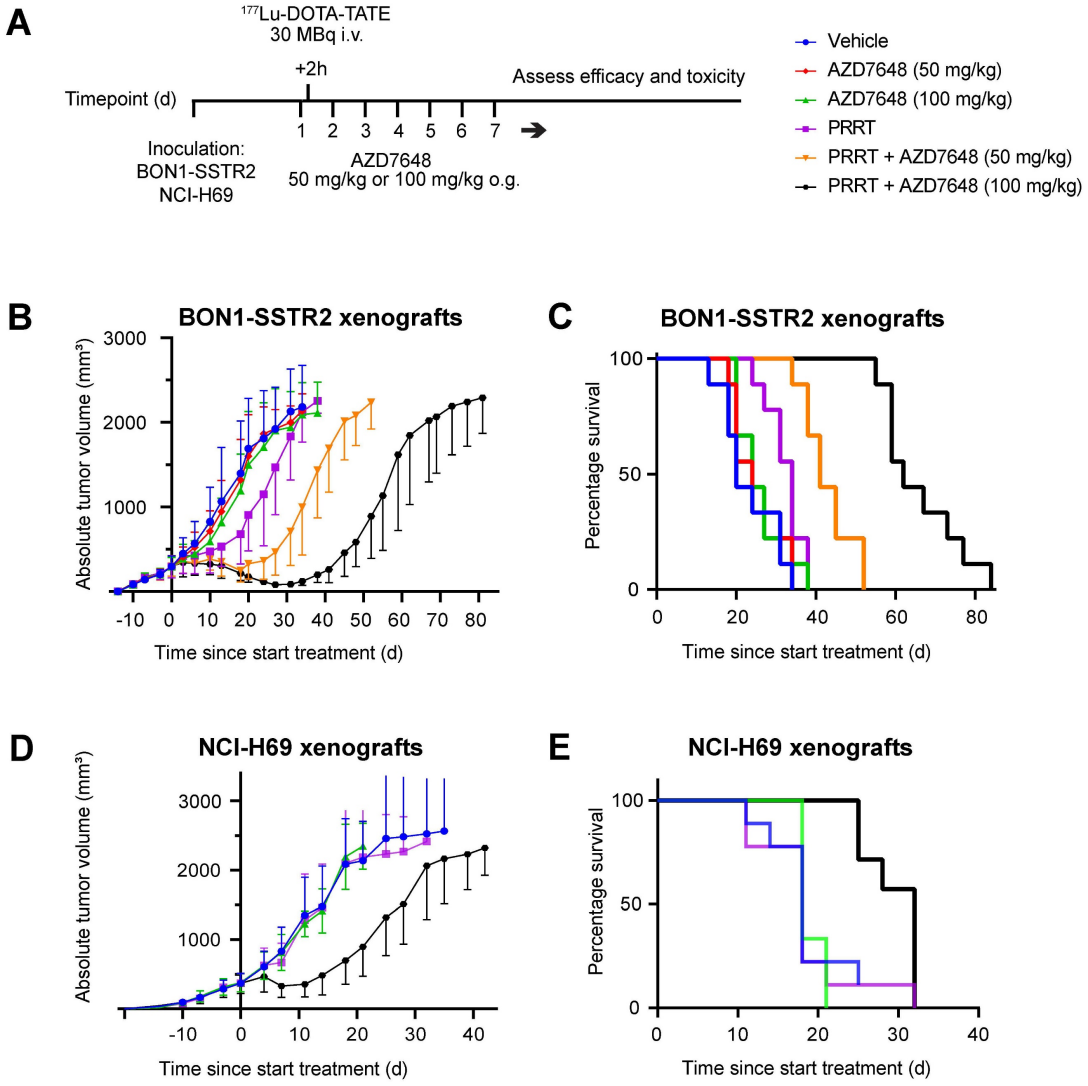
***In vivo* efficacy of PRRT + AZD7648 in xenografted mice. A,** Experimental setup for *in vivo* therapy study. After inoculation with BON1-SSTR2 or NCI-H69 cells and tumor growth, mice received a daily dose of AZD7648. At 2 h after the first AZD7648 administration, mice received a single dose of ^177^Lu-DOTA-TATE. **B,** Average absolute tumor volume (in mm^3^) for each treatment group of BON1-SSTR2 xenografted mice. Error bars represent SD. **C,** Kaplan-Meier curve visualizing survival of different treatment groups of BON1-SSTR2 xenografted mice.** D,** Average absolute tumor volume (in mm^3^) for each treatment group of NCI-H69 xenografted mice. AZD7648 was only administered at 100 mg/kg as this was the optimal dose in the BON1-SSTR2 xenografts. Error bars represent SD. **E,** Kaplan-Meier curve visualizing survival of different treatment groups of NCI-H69 xenografted mice.

To assess the efficacy and safety of combining PRRT and DNA-PKcs inhibition *in vivo*, BON1-SSTR2-xenografted mice were injected with a subtherapeutic dose of ^177^Lu-DOTA-TATE (30 MBq/mouse) and subjected to two different doses of AZD7648 (50 or 100 mg/kg/day for 7 consecutive days) (Figure 4A). Administered as monotherapy, both doses of AZD7648 did not have an effect on tumor growth, while PRRT monotherapy had a minor inhibitory effect (Figure 4B; Figure S5A). However, when PRRT was co-administered with AZD7648, a notable dose-dependent delay in tumor growth could be observed. Consequently, median survival in combination therapy groups increased to 41 days (PRRT + 50 mg/kg AZD7648) and 62 days (PRRT + 100 mg/kg AZD7648) after PRRT administration, compared to 34 days for PRRT monotherapy (Figure 4C). Importantly, none of the treatments had a significant effect on body weight, except for an individual mouse with severe weight loss in the 100 mg/kg AZD7648 monotherapy group, of which the cause is unknown (Figure S6).

PRRT combined with 100 mg/kg AZD7648 showed the highest efficacy for the BON1-SSTR2 tumors with no signs of acute toxicity, and we therefore tested this regimen in a second xenograft model with NCI-H69 tumors. No effect on tumor growth was seen for either AZD7648 or PRRT monotherapy, but the combination treatment resulted in strong tumor growth inhibition (Figure 4D, Figure S5B). Here, median survival after combination therapy increased to 32 days post-therapy initiation, compared to 18 days after either vehicle treatment, AZD7648 or PRRT monotherapy (Figure 4E).

After euthanization, BON1-SSTR2 xenografts were collected and analyzed for tissue morphology and SSTR2 expression. No morphological difference was observed between therapy groups and all analyzed tumors stained homogeneously positive for SSTR2 (Figure S7). This indicated that tumor recurrence after the initial regression upon administering PRRT + AZD7648 was not initiated from SSTR2-negative cells present in the tumor, but rather due to a different mechanism of therapy resistance.

### Combination treatment of PRRT and AZD7648 does not induce acute hematologic and renal toxicity

**Figure 5:**
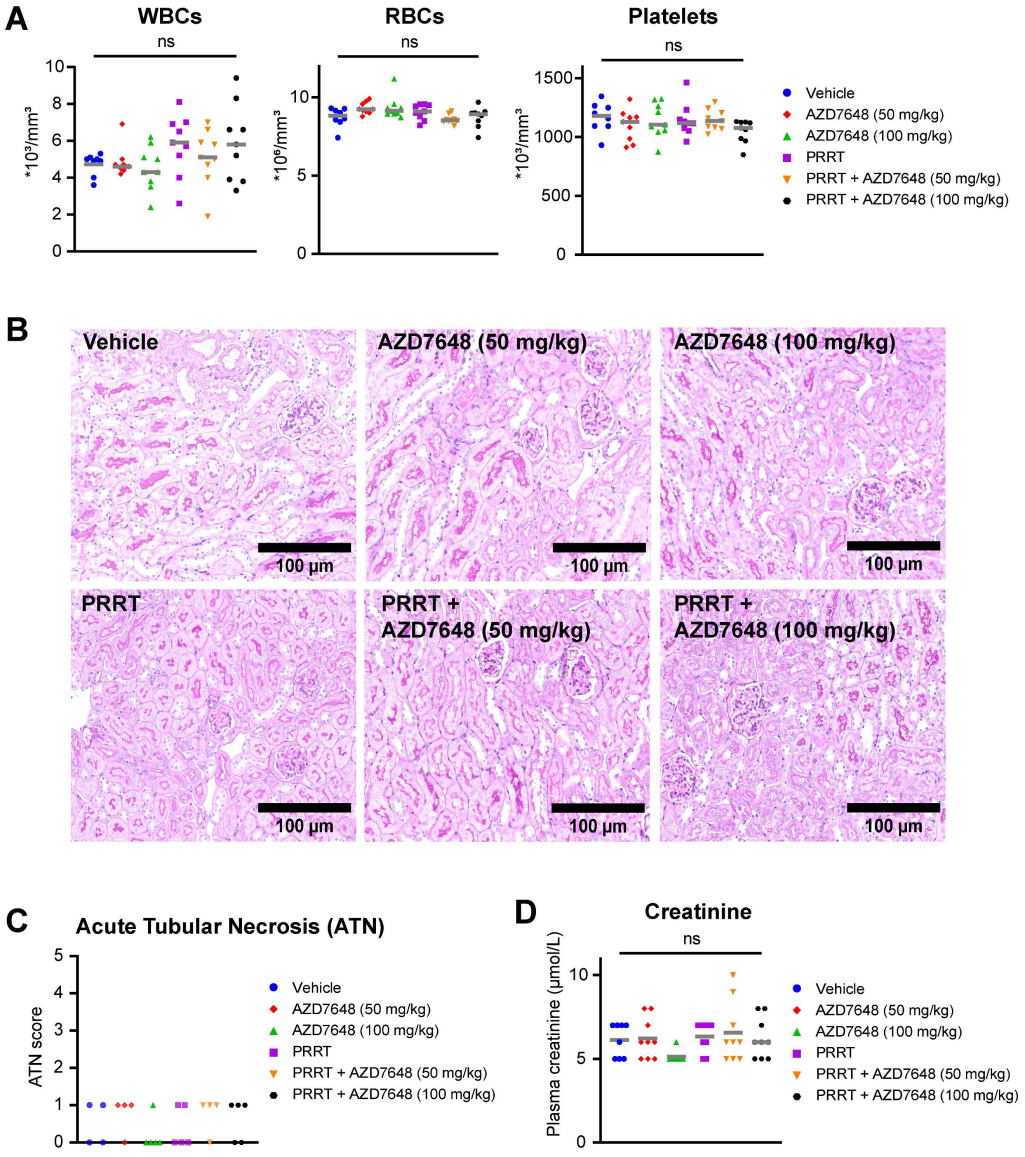
***In vivo* hematologic and renal toxicity of PRRT + AZD7648 in BON1-SSTR2 xenografted mice. A,** Whole blood assessment of the number white blood cells (WBCs), red blood cells (RBCs) and platelets for all individual mice in the different treatment groups. Grey horizontal bars represent the mean. **B,** Representative images (10x magnification) of PAS-stained kidney sections used for acute tubular necrosis (ATN) scoring.** C,** ATN scores for all treatment groups for at least 4 randomly selected mice per group. ATN was scored by an experienced nephropathologist by assessing tubular dilatation, cast deposition, brush border loss and necrosis. **D,** Determination of creatinine levels (in μmol/L) in blood plasma for all individuals in different treatment groups. Grey horizontal bars represent the mean.

As the bone marrow and kidneys are the main organs at risk during PRRT, we investigated whether co-treatment with AZD7648 would increase toxicity at these sites. For the mice bearing BON1-SSTR2 xenografts, hematologic toxicity was assessed at the timepoint of animal sacrifice by measuring white blood cell, red blood cell and platelet counts in whole blood samples (Figure 5A).

Both PRRT and AZD7648 monotherapy and PRRT + AZD7648 did not induce a significant difference in the level of any of these blood components compared to vehicle-treated mice, indicating absence of severe hematologic toxicity at the point of euthanization. For white blood cells, increased intra-group variability was seen after PRRT treatment (monotherapy and combination), but the cause of this is unknown. Subsequently, nephrotoxicity was assessed by scoring kidney histology for acute tubular necrosis (ATN) (Figure 5B-C). ATN scores were low in general (max. 1 on a 5-point scale) and similar between treatment groups. In addition, creatinine levels in blood plasma were determined (Figure 5D). No significant difference was observed for both PRRT monotherapy and combination therapy, compared to vehicle treatment. These data combined indicate absence of severe nephrotoxicity after any of the applied treatments at the timepoint of euthanization.

## Discussion

Interfering with the DDR machinery to sensitize cancer cells to ionizing radiation is a well-known strategy for EBRT, but evidence on combination regimens with PRRT is still lagging behind. Due to its central function in DSB repair, DNA-PKcs has been deemed a promising DDR target for this purpose. Importantly, in the past years various selective DNA-PKcs inhibitors have been developed, which have been tested extensively preclinically as monotherapy and in combination with ionizing radiation 19. Overall, targeting DNA-PKcs has been shown to significantly sensitize a plethora of cancer types *in vitro* and *in vivo* to different radiation types, such as photon and proton beams 17, 20-22, 29, 30.

Here, we showed strong and well-tolerated radiosensitization of PRRT in SSTR2-expressing preclinical models by the potent DNA-PKcs inhibitor AZD7648. This inhibitor targets DNA-PKcs kinase activity and exhibits high selectivity versus structurally related kinases such as ATM, ATR and PI3K isoforms, rendering it a suitable compound for potential clinical implementation 22. Our *in vitro* treatments in a panel of 3 SSTR2-expressing cell lines showed that AZD7648 dose-dependently and significantly reduced cell viability of PRRT-treated cells, as opposed to a relatively small effect of AZD7648 and PRRT monotherapy. This indicates a strong synergistic effect of PRRT and AZD7648, which was evident at submicromolar doses. In GOT1 and BON1-SSTR2 cell lines, two neuroendocrine tumor models with different genetic backgrounds, this was accompanied by a sharp increase in cell death compared to both monotherapies, indicating that at least part of the reduction in viability can be attributed to cancer-eradicating cellular fates such as apoptosis. The strong potentiation of PRRT by AZD7648 was confirmed in our *in vivo* xenograft experiments, as the selected doses of both AZD7648 and PRRT monotherapy had a minimal effect on tumor growth and survival, while combination therapy yielded significant tumor inhibition and corresponding increase in survival. However, especially in NCI-H69 xenografts, it was surprising that after PRRT monotherapy no difference in tumor control was detected compared to vehicle-treated mice, as previous *in vivo* experiments employing this model showed significant tumor-inhibitory activity of PRRT 6. When comparing SSTR2-expression of our NCI-H69 line with a different clonal lineage of the same cell type, our cell line showed, on average, a reduced SSTR2-expression by flow cytometric assessment (data not shown), most likely leading to lower PRRT uptake. Nevertheless, the magnitude of the combination therapy effect as compared to monotherapies indicates that a strong anti-tumor effect can be reached by combining subtherapeutic doses of PRRT and AZD7648. This means that patients with relative low level SSTR2-positive tumors that are not responding to PRRT could potentially benefit from this combination therapy regimen.

In GOT1 and BON1-SSTR2 cells, a significant increase in the number of DSBs was detected after combination therapy compared to PRRT monotherapy. This is in line with the central function of DNA-PKcs in the NHEJ pathway. However, both cell lines responded differently to the combination therapy. PRRT inhibited cell proliferation and induced a cell cycle block in GOT1 cells and this effect was increased upon combination therapy. In contrast, while PRRT had no detectable effect on BON1-SSTR2 nuclear size or cell cycle, these cells were unable to fully arrest cell cycle progression upon combination therapy, accompanied by polyploidy. It is likely that this is caused by therapy-induced endoreplication. It has been reported that p53 status is an important determinant for cellular effects after IR and DNA-PKcs inhibition, where p53 mutant cell lines were unable to induce cell cycle blocks and entered mitosis with high levels of DNA damage, leading to the formation of polyploid cells 31. Indeed, the wildtype BON1 model has been reported to harbor a mutation in the p53 gene 32. Consistently, for GOT1, a p53 wildtype model 32, no effect on nuclear size was detected. In addition, it has been shown that p53 status influences cellular fate after ionizing radiation and DNA-PKcs inhibitor treatment, with the induction of premature senescence in p53 wildtype cells and apoptosis in p53 mutant cells 31. However, in our experiments we detected significant cell death induction both in GOT1 and BON1-SSTR2 after PRRT + AZD7648, meaning that, although different cellular response mechanisms are involved, eventually cell death is induced in both p53 wildtype and mutant cells. The latter might be highly dependent on specific, yet unidentified cellular characteristics.

Importantly, in the case of p53 mutant cells, induction of polyploidy by endoreplication has been debated to be a potential resistance mechanism for tumors after therapy 33. In addition, it has been shown that polyploid cells rewire their DDR to cope with cellular stresses during endoreplication 34. In this study, under AZD7648 treatment we detected high levels of unresolved DSBs in polyploid nuclei, indicating that DNA-PKcs inhibition might exceed the threshold of cellular stress in these cells and consequently induces cell death. For example, it is likely that these treated cells are subjected to high levels of replication stress after endoreplication. When analyzing tumor morphology after PRRT + AZD7648 in our *in vivo* studies, the presence of large polyploid nuclei was not detected. This leaves the question if the observed endoreplication *in vitro* also occurs *in vivo* and if yes, these cells could not be detected because they had already been killed after therapy. Nevertheless, BON1 has been shown to harbor multiple other genomic lesions in genes associated with cell cycle progression, such as *CDKN2A* and *CHEK2* 32, which also might play important roles in the formation of polyploid cells after PRRT + AZD7648. In addition, it must be noted that mutations in the p53 gene are rare in NETs 35, which is a limitation of using BON1-SSTR2 for preclinical studies for this specific tumor type. Thus, exact genomic determinants of therapy response and (long-term) consequences for tumor control remain unclear and require additional research.

In addition to the function of DNA-PKcs in DSB repair, other cellular roles of DNA-PKcs might play a role in the described radiosensitizing effects. For example, it has been shown that DNA-PKcs is involved in mitotic progression, which might add to the observed phenotype after PRRT + AZD7648 in BON1-SSTR2 cells 36. However, another possibility is that these effects are the result of an impaired DNA damage response upon AZD648 treatment and elevated levels of DNA damage *per se* and not of DNA repair-independent functions of DNA-PKcs, which warrants further investigation.

One of the major concerns when combining ionizing radiation and DDR inhibitors is overlapping toxicity profiles 37. Specifically for DNA-PKcs inhibitors, systemic administration might lead to the impairment of radiation-induced DSB repair in healthy tissues throughout the body. In our xenograft studies, we assessed hematologic and renal toxicity as these are the main dose-limiting organs for PRRT and DNA-PKcs inhibition could potentially increase PRRT-induced damage here 2, 3. These experiments indicated that combination of PRRT and AZD7648 induced no severe acute toxicity at these sites. Previous research on combining radiation with AZD7648 showed increased normal tissue toxicity after EBRT and AZD7648 combination therapy *in vivo*, along with significant body weight loss 38. Not only are the non-target organs at risk different between EBRT and PRRT, but also a different toxicity profile can be expected based on differences in radiobiological characteristics. Compared to EBRT, a higher tumor selectivity of PRRT might render a more favorable toxicity profile to combine with systemic DNA-PKcs inhibition. Moreover, the total administered radiation dose between the aforementioned study assessing AZD7648 with EBRT and our study are not matched. Nevertheless, a drawback of our approach might be that we only assessed acute toxicity at a single timepoint upon euthanization. In addition, the use of the immunodeficient mouse strain Rj:NMRI-*Foxn1^nu/nu^* limits conclusions that can be drawn on therapy-induced lymphotoxicity. However, the fact that we did not detect any signs of acute hematologic and renal toxicity, along with no significant effects on body weight along the course of the experiment, strongly supports the hypothesis that combining PRRT and AZD7648 in this regimen is well-tolerated in mice. In the future, long-term studies are needed to investigate late toxicity effects, most importantly on kidneys and bone marrow, as well as the effect of different combination therapy scheduling on efficacy and toxicity. For example, the effect of starting DNA-PKcs inhibitor treatment after PRRT administration, when the radionuclide has been cleared from the dose-limiting organs but is still present in the tumor, might be considered.

Previous work by us and others has evaluated other combinatorial strategies of DDR inhibitors and PRRT, such as poly (ADP-ribose) polymerase (PARP)-inhibition 9, 39, 40. Here, inhibitors of PARP1/2 were used to sensitize several SSTR2-expressing cell lines to PRRT. Inhibition of PARP1/2 attenuates its role in single strand break repair and these breaks can be converted to DSBs during DNA replication 41. In this way, PARP1/2 inhibition will preferentially target replicating cells, while DNA-PKcs inhibition targets NHEJ as the major DSB repair pathway after IR, which is active throughout the cell cycle. Compared to PARP1/2 inhibition, DNA-PKcs inhibition thus might show better efficacy in slow-growing tumors, which is often the case for NETs, especially those of lower grades.

In conclusion, we have shown strong and tolerable potentiation of PRRT by DNA-PKcs inhibitor AZD7648 in preclinical neuroendocrine tumor *in vitro* and *in vivo* models. Further research on dosing and scheduling of PRRT co-administrated with new generation DNA-PKcs inhibitors such as AZD7648, along with additional toxicity studies, should pave the way to clinical trials in humans, as this might be very different from combination therapies with EBRT. Currently, DNA-PKcs inhibitors are evaluated as monotherapy in advanced clinical trials, showing good tolerability but limited efficacy 19. In addition, a few clinical trials are underway evaluating the effects of DNA-PKcs inhibition and EBRT, and results on efficacy and safety in humans are expected soon (e.g. NCT05116254, NCT04555577, NCT04068194). The development of next generation DNA-PKcs inhibitors could potentially further improve therapy outcomes. This might include inhibitors with a different mechanism of action, such as blocking specific interaction sites of DNA-PKcs and downstream proteins, enabling targeting of specific DNA-PKcs functions important in tumor progression after ionizing radiation. Furthermore, combination of DNA-PKcs inhibitors with α-emitters might yield significantly enhanced responses, considering the increased probability of DSB induction compared to β-emitters such as lutetium-177 42. These developments, together with an improved understanding of PRRT radiobiology, hold the promise to stimulate implementation of PRRT-based combination regimens in the clinic and thus further improve responses to radionuclide therapies in neuroendocrine tumors and other cancer types.

## Supplementary Material

Supplementary methods and figures.

## Abbreviations

ATN: acute tubular necrosis
C-NHEJ: classical non-homologous end joining
DDR: DNA damage response
DNA-PKcs: DNA-dependent protein kinase catalytic subunit
DSB: double-strand break
EBRT: external beam radiotherapy
EdU: 5-ethynyl-2'-deoxyuridine
H&E: hematoxylin & eosin
HBSS: Hank's Balanced Salt Solution
IF: immunofluorescence
IHC: immunohistochemistry
MMEJ: microhomology-mediated end joining
NET: neuroendocrine tumor
NHEJ: non-homologous end joining
PARP: poly (ADP-ribose) polymerase
PAS: periodic acid-Schiff
PRRT: peptide receptor radionuclide therapy
SSTR2: somatostatin receptor subtype 2

## References

[1] Hofland J, Kaltsas G, de Herder WW (2020). Advances in the Diagnosis and Management of Well-Differentiated Neuroendocrine Neoplasms. Endocr Rev.

[2] Bodei L, Kidd M, Paganelli G, Grana CM, Drozdov I, Cremonesi M (2015). Long-term tolerability of PRRT in 807 patients with neuroendocrine tumours: the value and limitations of clinical factors. Eur J Nucl Med Mol Imaging.

[3] Strosberg J, El-Haddad G, Wolin E, Hendifar A, Yao J, Chasen B (2017). Phase 3 Trial of (177)Lu-Dotatate for Midgut Neuroendocrine Tumors. N Engl J Med.

[4] Terry SYA, Nonnekens J, Aerts A, Baatout S, de Jong M, Cornelissen B (2019). Call to arms: need for radiobiology in molecular radionuclide therapy. Eur J Nucl Med Mol Imaging.

[5] Kam BL, Teunissen JJ, Krenning EP, de Herder WW, Khan S, van Vliet EI (2012). Lutetium-labelled peptides for therapy of neuroendocrine tumours. Eur J Nucl Med Mol Imaging.

[6] Feijtel D, Doeswijk GN, Verkaik NS, Haeck JC, Chicco D, Angotti C (2021). Inter and intra-tumor somatostatin receptor 2 heterogeneity influences peptide receptor radionuclide therapy response. Theranostics.

[7] Ciccia A, Elledge SJ (2010). The DNA damage response: making it safe to play with knives. Mol Cell.

[8] Chan TG, O'Neill E, Habjan C, Cornelissen B (2020). Combination Strategies to Improve Targeted Radionuclide Therapy. J Nucl Med.

[9] Nonnekens J, van Kranenburg M, Beerens CE, Suker M, Doukas M, van Eijck CH (2016). Potentiation of Peptide Receptor Radionuclide Therapy by the PARP Inhibitor Olaparib. Theranostics.

[10] Pouget JP, Lozza C, Deshayes E, Boudousq V, Navarro-Teulon I (2015). Introduction to radiobiology of targeted radionuclide therapy. Front Med (Lausanne).

[11] Chapman JR, Taylor MR, Boulton SJ (2012). Playing the end game: DNA double-strand break repair pathway choice. Mol Cell.

[12] Hendrickson EA, Qin XQ, Bump EA, Schatz DG, Oettinger M, Weaver DT (1991). A link between double-strand break-related repair and V(D)J recombination: the scid mutation. Proc Natl Acad Sci U S A.

[13] Blunt T, Gell D, Fox M, Taccioli GE, Lehmann AR, Jackson SP (1996). Identification of a nonsense mutation in the carboxyl-terminal region of DNA-dependent protein kinase catalytic subunit in the scid mouse. Proc Natl Acad Sci U S A.

[14] Taccioli GE, Amatucci AG, Beamish HJ, Gell D, Xiang XH, Torres Arzayus MI (1998). Targeted disruption of the catalytic subunit of the DNA-PK gene in mice confers severe combined immunodeficiency and radiosensitivity. Immunity.

[15] Beskow C, Skikuniene J, Holgersson A, Nilsson B, Lewensohn R, Kanter L (2009). Radioresistant cervical cancer shows upregulation of the NHEJ proteins DNA-PKcs, Ku70 and Ku86. Br J Cancer.

[16] Bouchaert P, Guerif S, Debiais C, Irani J, Fromont G (2012). DNA-PKcs expression predicts response to radiotherapy in prostate cancer. Int J Radiat Oncol Biol Phys.

[17] Noguchi T, Shibata T, Fumoto S, Uchida Y, Mueller W, Takeno S (2002). DNA-PKcs expression in esophageal cancer as a predictor for chemoradiation therapeutic sensitivity. Ann Surg Oncol.

[18] Mohiuddin IS, Kang MH (2019). DNA-PK as an Emerging Therapeutic Target in Cancer. Front Oncol.

[19] Hu S, Hui Z, Lirussi F, Garrido C, Ye XY, Xie T (2021). Small molecule DNA-PK inhibitors as potential cancer therapy: a patent review (2010-present). Expert Opin Ther Pat.

[20] Ciszewski WM, Tavecchio M, Dastych J, Curtin NJ (2014). DNA-PK inhibition by NU7441 sensitizes breast cancer cells to ionizing radiation and doxorubicin. Breast Cancer Res Treat.

[21] Zenke FT, Zimmermann A, Sirrenberg C, Dahmen H, Kirkin V, Pehl U (2020). Pharmacologic Inhibitor of DNA-PK, M3814, Potentiates Radiotherapy and Regresses Human Tumors in Mouse Models. Mol Cancer Ther.

[22] Fok JHL, Ramos-Montoya A, Vazquez-Chantada M, Wijnhoven PWG, Follia V, James N (2019). AZD7648 is a potent and selective DNA-PK inhibitor that enhances radiation, chemotherapy and olaparib activity. Nat Commun.

[23] Dalm SU, Nonnekens J, Doeswijk GN, de Blois E, van Gent DC, Konijnenberg MW (2016). Comparison of the Therapeutic Response to Treatment with a 177Lu-Labeled Somatostatin Receptor Agonist and Antagonist in Preclinical Models. J Nucl Med.

[24] Weterings E, Verkaik NS, Bruggenwirth HT, Hoeijmakers JH, van Gent DC (2003). The role of DNA dependent protein kinase in synapsis of DNA ends. Nucleic Acids Res.

[25] Snijders MLH, Zajec M, Walter LAJ, de Louw R, Oomen MHA, Arshad S (2019). Cryo-Gel embedding compound for renal biopsy biobanking. Sci Rep.

[26] Grozinsky-Glasberg S, Shimon I, Rubinfeld H (2012). The role of cell lines in the study of neuroendocrine tumors. Neuroendocrinology.

[27] Chen BP, Chan DW, Kobayashi J, Burma S, Asaithamby A, Morotomi-Yano K (2005). Cell cycle dependence of DNA-dependent protein kinase phosphorylation in response to DNA double strand breaks. J Biol Chem.

[28] Panier S, Boulton SJ (2014). Double-strand break repair: 53BP1 comes into focus. Nat Rev Mol Cell Biol.

[29] Doherty RE, Bryant HE, Valluru MK, Rennie IG, Sisley K (2019). Increased Non-Homologous End Joining Makes DNA-PK a Promising Target for Therapeutic Intervention in Uveal Melanoma. Cancers (Basel).

[30] Timme CR, Rath BH, O'Neill JW, Camphausen K, Tofilon PJ (2018). The DNA-PK Inhibitor VX-984 Enhances the Radiosensitivity of Glioblastoma Cells Grown *In vitro* and as Orthotopic Xenografts. Mol Cancer Ther.

[31] Sun Q, Guo Y, Liu X, Czauderna F, Carr MI, Zenke FT (2019). Therapeutic Implications of p53 Status on Cancer Cell Fate Following Exposure to Ionizing Radiation and the DNA-PK Inhibitor M3814. Mol Cancer Res.

[32] Hofving T, Arvidsson Y, Almobarak B, Inge L, Pfragner R, Persson M (2018). The neuroendocrine phenotype, genomic profile and therapeutic sensitivity of GEPNET cell lines. Endocr Relat Cancer.

[33] Shu Z, Row S, Deng WM (2018). Endoreplication: The Good, the Bad, and the Ugly. Trends Cell Biol.

[34] Zheng L, Dai H, Zhou M, Li X, Liu C, Guo Z (2012). Polyploid cells rewire DNA damage response networks to overcome replication stress-induced barriers for tumour progression. Nat Commun.

[35] Lohmann DR, Funk A, Niedermeyer HP, Haupel S, Hofler H (1993). Identification of p53 gene mutations in gastrointestinal and pancreatic carcinoids by nonradioisotopic SSCA. Virchows Arch B Cell Pathol Incl Mol Pathol.

[36] Huang B, Shang ZF, Li B, Wang Y, Liu XD, Zhang SM (2014). DNA-PKcs associates with PLK1 and is involved in proper chromosome segregation and cytokinesis. J Cell Biochem.

[37] Pilie PG, Tang C, Mills GB, Yap TA (2019). State-of-the-art strategies for targeting the DNA damage response in cancer. Nat Rev Clin Oncol.

[38] Hong CR, Buckley CD, Wong WW, Anekal PV, Dickson BD, Bogle G (2022). Radiosensitisation of SCCVII tumours and normal tissues in mice by the DNA-dependent protein kinase inhibitor AZD7648. Radiother Oncol.

[39] Feijtel D, Reuvers TGA, van Tuyll-van Serooskerken C, de Ridder CMA, Stuurman DC, de Blois E (2023). *In vivo* Efficacy Testing of Peptide Receptor Radionuclide Therapy Radiosensitization Using Olaparib. Cancers (Basel).

[40] Purohit NK, Shah RG, Adant S, Hoepfner M, Shah GM, Beauregard JM (2018). Potentiation of (177)Lu-octreotate peptide receptor radionuclide therapy of human neuroendocrine tumor cells by PARP inhibitor. Oncotarget.

[41] Curtin NJ, Szabo C (2013). Therapeutic applications of PARP inhibitors: anticancer therapy and beyond. Mol Aspects Med.

[42] Brons S, Jakob B, Taucher-Scholz G, Kraft G (2001). Heavy ion production of single- and double-strand breaks in plasmid DNA in aqueous solution. Phys Med.

