# Supplementary material for: DNA-PKcs inhibitors sensitize neuroendocrine tumor cells to peptide receptor radionuclide therapy in vitro and in vivo
Source: Theranostics. 2023 May 21;13(10):3117–30. doi: 10.7150/thno.82963 (PMC10283055; doi:10.7150/thno.82963)
Supplement: Supplementary file 1 — Supplementary methods and figures. [file thnov13p3117s1.pdf]

Supplementary materials

SSTR2-expression analysis

SSTR2-expression was assessed in BON1 and BON1-SSTR2 cells by flow cytometry. For each cell type, $1x10^6$ cells were fixed in 70% ethanol in suspension and incubated with primary antibody in PBS + 1% BSA for 1 h at RT. Cells were washed in PBS + 1% BSA, after which they were incubated with secondary antibody in PBS + 1% BSA for 1 h at RT in the dark. Cells were washed twice and resuspended in 1 µg/mL DAPI in PBS until analysis. SSTR2- and DAPI-intensities were recorded on a LSRFortessa flow cytometer. Data analysis was done using FlowJo™ software. Experiments were performed as 2 independent replicates.

Uptake experiments

For assessment of membrane-bound and internalized fractions, $2x10^5$ BON1 or BON1-SSTR2 cells were plated in triplicate in 24-well plates and treated with ^{177}Lu-DOTA-TATE ($1.9x10^{-8}$ M, 1 MBq/mL) in culture medium for 2 h at 37°C. Cells were washed two times with PBS and the membrane-bound fraction was collected by incubation in 50 mM glycine + 100 mM NaCl (pH 2.8) for 10 minutes at RT. Cells were washed with PBS and this fraction was pooled with the previous collected fraction. Hereafter, cells were lysed with 0.1 M NaOH for 5 minutes at RT and washed twice with PBS. These fractions were pooled and analyzed as the internalized fraction. Activity was measured using a 1480 WIZARD automatic γ-counter (Perkin Elmer). Experiments were performed as 3 independent replicates.

End-joining assay

Assessment of end-joining activity was done as described before [1]. In short, BON1-SSTR2 cells were grown in 6-well plates to approximately 50% confluency and treated with vehicle or

1 µM AZD7648. After 24 h, cells were transfected with linearized pDVG94 plasmid DNA using Lipofectamine (Invitrogen). At day 2 after transfection, extrachromosomal DNA was isolated from cell cultures and intracellularly joined pDVG94 DNA was PCR-amplified. Amplified product was digested by the restriction enzyme BstXI. The resulting products were separated on polyacrylamide gel in TBE buffer and visualized by ethidium bromide. Quantification of the shift from C-NHEJ to MMEJ was done using FIJI.

Western blotting

BON1-SSTR2 cells were treated with ^{177}Lu-DOTA-TATE (1.9x10^{-8} M, 1 MBq/mL) or vehicle in suspension (2x10^{5} cells/mL in culture medium) and either lysed directly after incubation or plated in 6-well plates in presence of 1 µM AZD7648 or vehicle and lysed 24 h post-PRRT. Protein lysates were prepared in Laemmli sample buffer. Subsequently, 10 µg protein was loaded per lane on Mini-PROTEAN TGX Precast gels (Biorad) and gel electrophoresis was run for 1.5 h at 100V. Proteins were transferred to Immobilon-P PVDF membranes (Merck) for 2 h at 100V on ice. Membranes were blocked in PBS + 3% skimmed milk (Merck Millipore) and incubated with primary antibody in blocking buffer at 4 ºC overnight. Subsequently, membranes were washed in PBS + 0.1% Tween-20 (Sigma) and incubated with secondary antibody for 1 h at RT. Protein bands were detected by electrochemiluminescence on an Amersham Imager 600 (GE Healthcare Life Sciences). Experiments were performed as 2 independent replicates.

References

1. Verkaik, N.S.; Esveldt-van Lange, R.E.; van Heemst, D.; Bruggenwirth, H.T.; Hoeijmakers, J.H.; Zdzienicka, M.Z.; van Gent, D.C. Different types of V(D)J recombination and end-joining defects in DNA double-strand break repair mutant mammalian cells. Eur J Immunol. 2002; 32: 701-709.

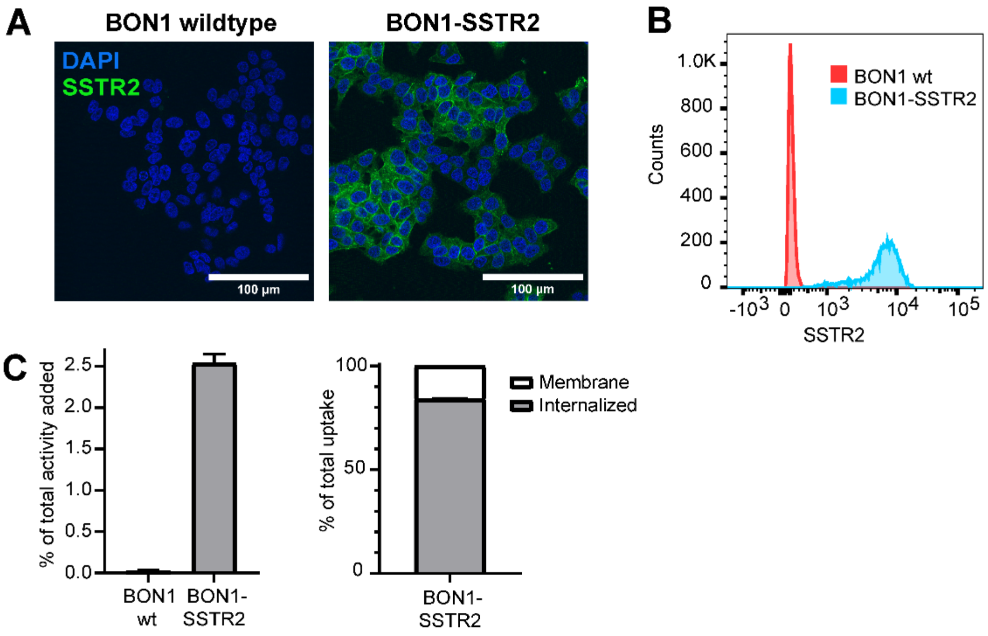

Supplementary figure 1. Characterization of BON1-SSTR2 cell line. A, IF-staining for SSTR2 (green) for BON1 wildtype vs. BON1-SSTR2 cells. DAPI (blue) was used as nuclear counterstain. **B,** Assessment of SSTR2-expression in BON1 wildtype vs. BON1-SSTR2 cells by flow cytometry. **C, Left,** Total uptake expressed as % of total activity added of ^{177}Lu-DOTA-TATE in BON1 wildtype vs. BON1-SSTR2 cells (left). **Right,** Uptake of ^{177}Lu-DOTA-TATE in BON1-SSTR2 cells analyzed separately as membrane-bound and internalized fraction.

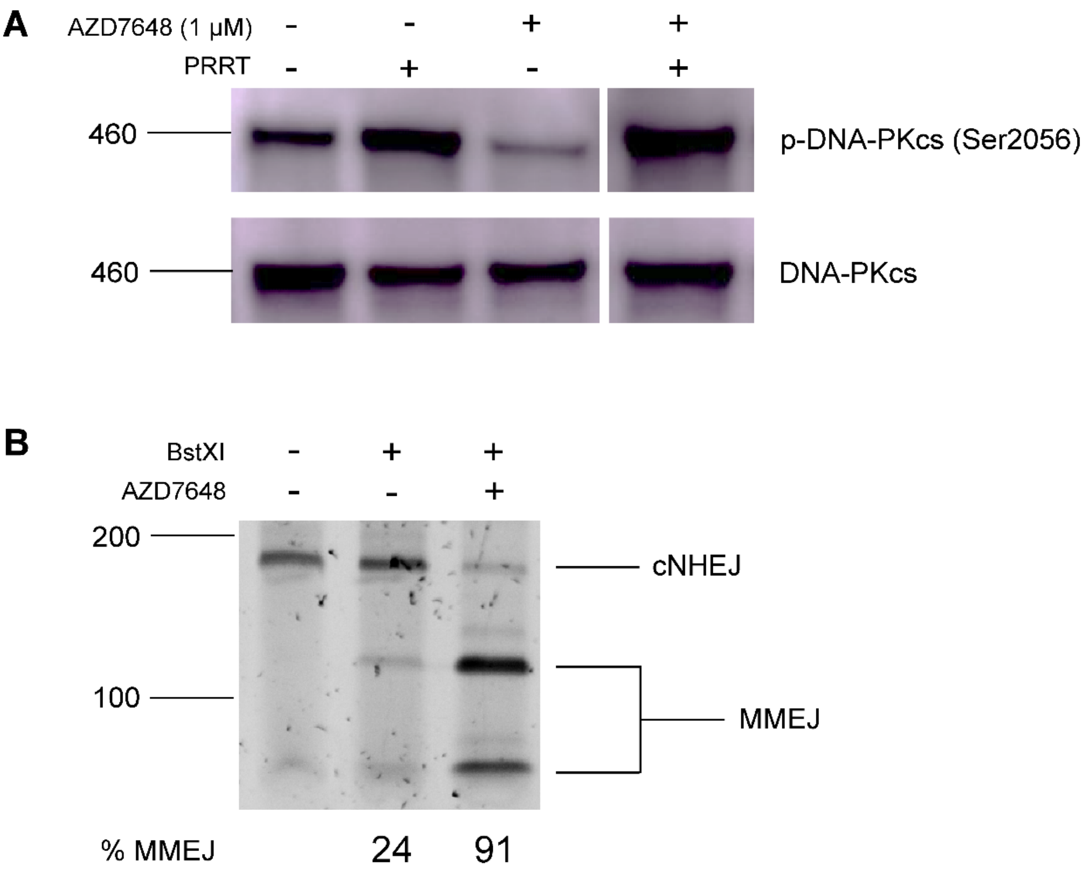

Supplementary figure 2. Validation of inhibition of DNA-PKcs catalytic activity by AZD7648 and effects on NHEJ levels in BON1-SSTR2 cells. A, Western blot analysis of DNA-PKcs activation levels (Ser2056 autophosphorylation) after AZD7648 and/or PRRT. Total DNA-PKcs levels were used as a loading control. **B,** Assessment of the balance between C-NHEJ and MMEJ repair pathways upon treatment with 1 µM AZD7648 by end-joining assay. Vehicle-treated cells (middle lane) were used as negative control. Undigested PCR product (left lane) was used as an internal control.

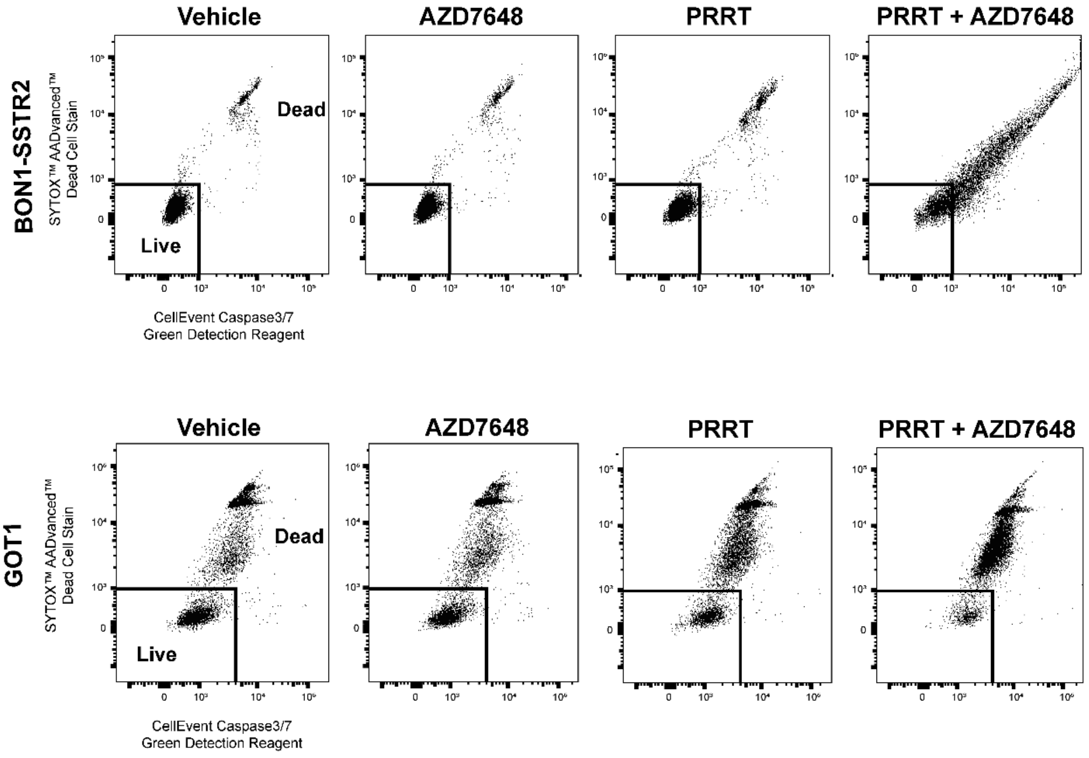

Supplementary figure 3. Representative scatter plots of flow cytometric cell death analysis. Plots are shown for GOT1 (upper panel) and BON1-SSTR2 (lower panel) for vehicle-, AZD7648-, PRRT- and combination therapy-treated cells. The percentage of dead cells is determined by analyzing CellEvent Caspase3/7 Green Detection Reagent (x-axis) and SYTOX™ AADvanced™ Dead Cell Stain (y-axis) signals. Gating strategy is indicated in vehicle treatment plots.

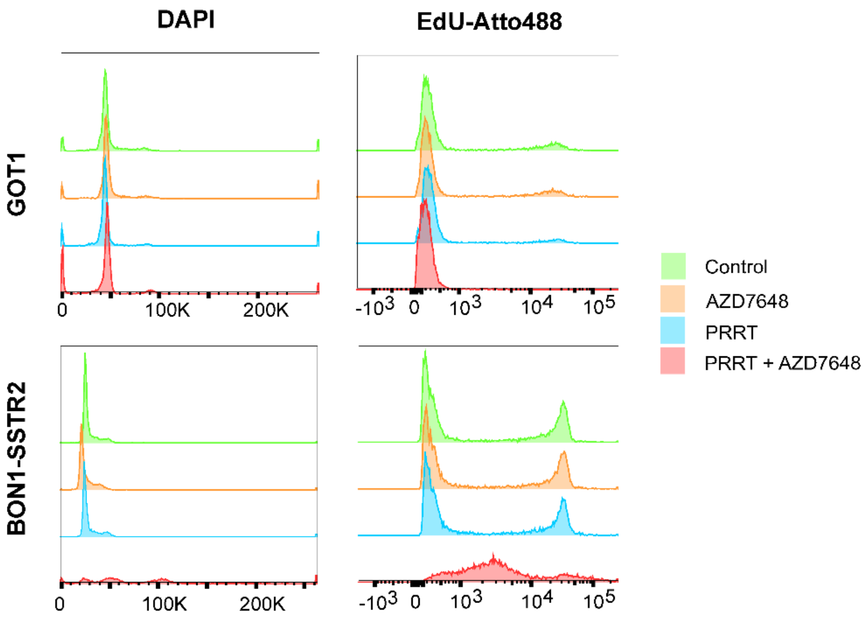

Supplementary figure 4. Representative histograms for cell cycle distribution analysis for GOT1 (upper panel) and BON1-SSTR2 (lower panel). Histograms are shown for DAPI- (left) and EdU-Atto488-signal (right).

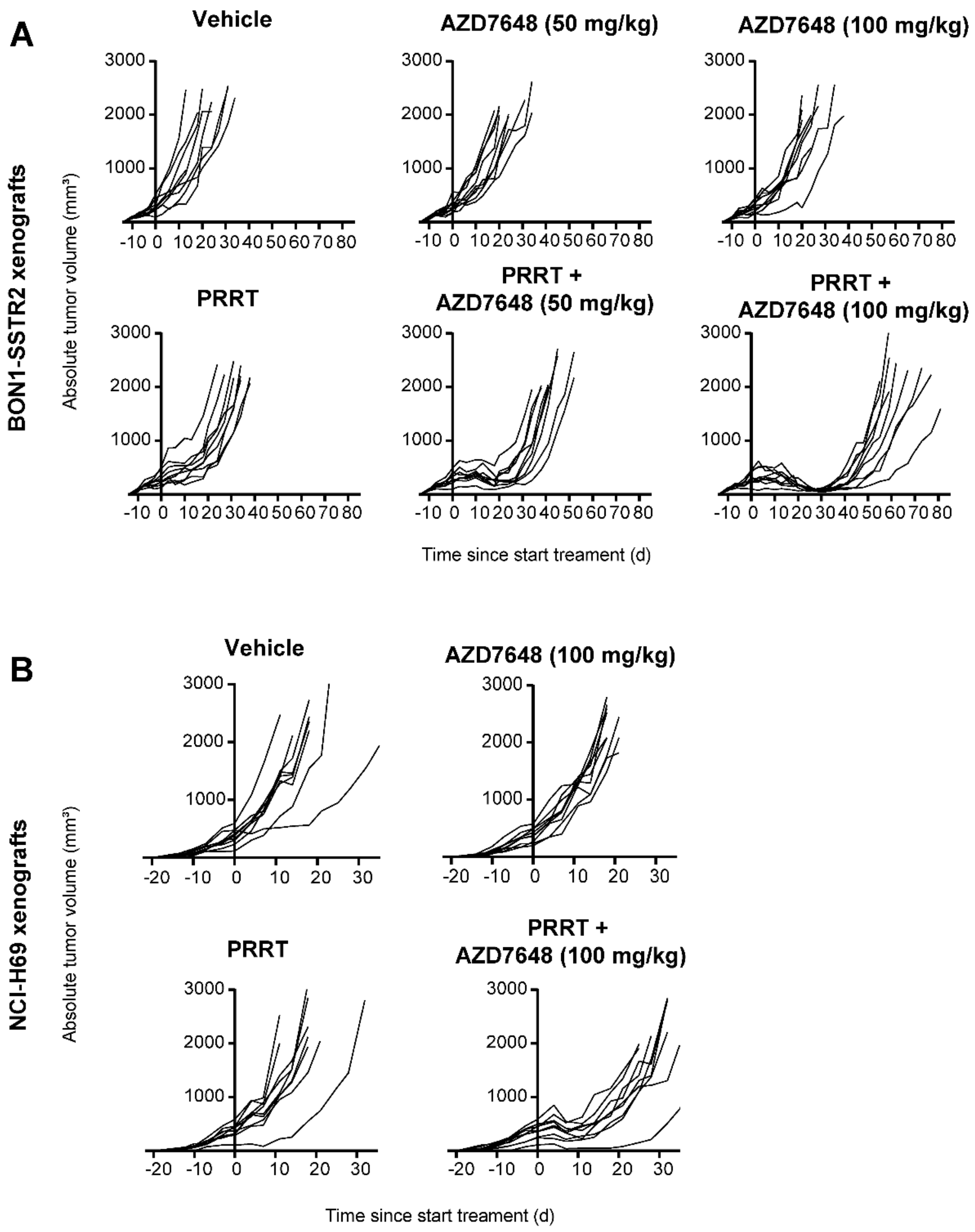

Supplementary figure 5. Individual tumor growth curves (absolute tumor volume) for BON1-SSTR2 (A) and NCI-H69 (B) xenografts.

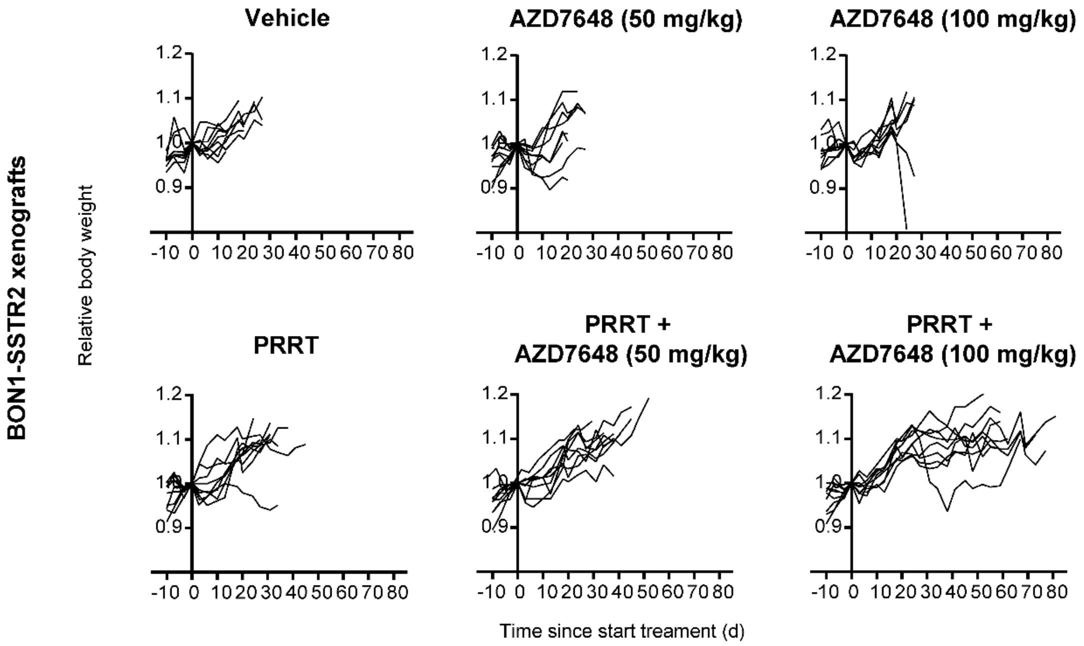

Supplementary figure 6. Relative body weights of individual mice per treatment group for BON1-SSTR2 xenografts.

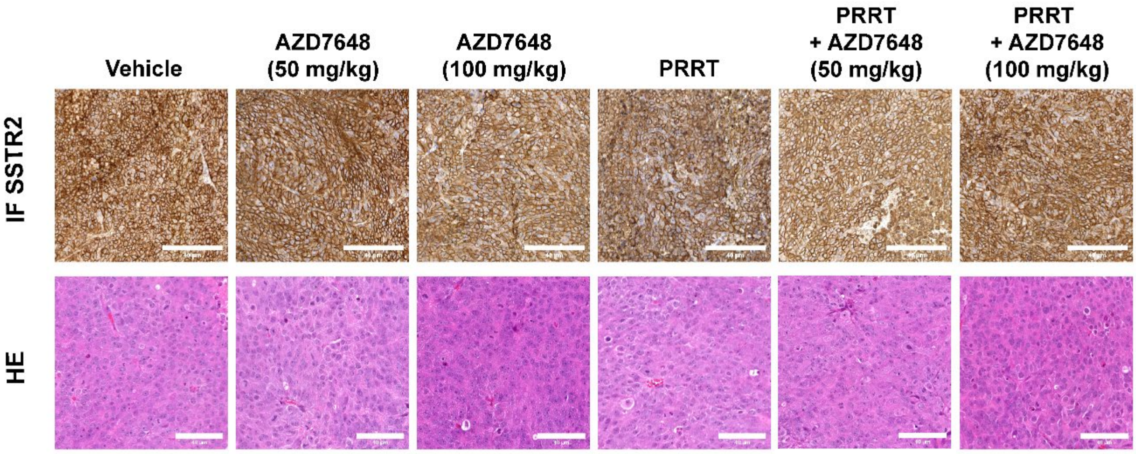

Supplementary figure 7. Analysis of SSTR2-expression levels (upper panel) and tumor morphology (lower panel) in BON1-SSTR2 xenografts. Tumors were collected and analyzed for SSTR2 by IHC and morphology by H&E staining. One representative tumor is shown per treatment group.
